# Concept and design of a genome-wide association genotyping array tailored for transplantation-specific studies

**DOI:** 10.1186/s13073-015-0211-x

**Published:** 2015-10-01

**Authors:** Yun R. Li, Jessica van Setten, Shefali S. Verma, Yontao Lu, Michael V. Holmes, Hui Gao, Monkol Lek, Nikhil Nair, Hareesh Chandrupatla, Baoli Chang, Konrad J. Karczewski, Chanel Wong, Maede Mohebnasab, Eyas Mukhtar, Randy Phillips, Vinicius Tragante, Cuiping Hou, Laura Steel, Takesha Lee, James Garifallou, Toumy Guettouche, Hongzhi Cao, Weihua Guan, Aubree Himes, Jacob van Houten, Andrew Pasquier, Reina Yu, Elena Carrigan, Michael B. Miller, David Schladt, Abdullah Akdere, Ana Gonzalez, Kelsey M. Llyod, Daniel McGinn, Abhinav Gangasani, Zach Michaud, Abigail Colasacco, James Snyder, Kelly Thomas, Tiancheng Wang, Baolin Wu, Alhusain J. Alzahrani, Amein K. Al-Ali, Fahad A. Al-Muhanna, Abdullah M. Al-Rubaish, Samir Al-Mueilo, Dimitri S. Monos, Barbara Murphy, Kim M. Olthoff, Cisca Wijmenga, Teresa Webster, Malek Kamoun, Suganthi Balasubramanian, Matthew B. Lanktree, William S. Oetting, Pablo Garcia-Pavia, Daniel G. MacArthur, Paul I W de Bakker, Hakon Hakonarson, Kelly A. Birdwell, Pamala A. Jacobson, Marylyn D. Ritchie, Folkert W. Asselbergs, Ajay K. Israni, Abraham Shaked, Brendan J. Keating

**Affiliations:** Medical Scientist Training Program, Perelman School of Medicine, University of Pennsylvania, Philadelphia, PA USA; The Children’s Hospital of Philadelphia, Philadelphia, PA USA; Department of Cardiology, Division of Heart and Lungs, University Medical Center Utrecht, Utrecht, The Netherlands; Affymetrix Incorporated, Santa Clara, CA USA; Penn Transplant Institute, Hospital of the University of Pennsylvania, Philadelphia, PA USA; Center for Systems Genomics, The Pennsylvania State University, University Park, PA USA; Analytic and Translational Genetics Unit, Massachusetts General Hospital, Boston, MA USA; Program in Medical and Population Genetics, Broad Institute of Harvard and MIT, Cambridge, MA USA; Department of Medical Genetics, Center for Molecular Medicine and Department of Epidemiology, Julius Center for Health Sciences and Primary Care, University Medical Center Utrecht, Utrecht, The Netherlands; BGI-Shenzhen, Shenzhen, China; Department of Biology, University of Copenhagen, Copenhagen, Denmark; Division of Biostatistics, University of Minnesota, Minneapolis, MN USA; Department of Psychology, University of Minnesota, Minneapolis, MN USA; College of Medicine, University of Dammam, Dammam, Kingdom of Saudi Arabia; Department of Pathology and Laboratory Medicine, Perelman School of Medicine, University of Pennsylvania and the Children’s Hospital of Philadelphia, Philadelphia, PA USA; Division of Nephrology and Department of Medicine, Icahn School of Medicine at Mount Sinai, New York, NY USA; Department of Genetics, The University Medical Center Groningen, Groningen, The Netherlands; Program in Computational Biology and Bioinformatics, and Molecular Biophysics and Biochemistry Department, Yale University, New Haven, CT 06520 USA; Experimental and Clinical Pharmacology, University of Minnesota, Minneapolis, MN USA; Heart Failure and Inherited Cardiac Diseases Unit, Department of Cardiology, Hospital Universitario Puerta de Hierro Majadahonda, Madrid, Spain; School of Medicine, Vanderbilt University, Nashville, TN USA; College of Pharmacy, University of Minnesota, Minneapolis, USA; Durrer Center for Cardiogenetic Research, ICIN-Netherlands Heart Institute, Utrecht, The Netherlands; Institute of Cardiovascular Science, faculty of Population Health Sciences, University College London, London, UK; Department of Clinical Laboratories Sciences, College of Applied Medical Sciences, King Saud University, Riyadh, Saudi Arabia; Minneapolis Medical Research Foundation, Hennepin County Medical Center, Minneapolis, MN USA; Hennepin County Medical Center, University of Minneosta, Minneapolis, MN USA; Department of Pediatrics, University of Pennsylvania, Philadelphia, PA USA; Division of Transplantation, 2 Dulles, Hospital of the University of Pennsylvania, 3400 Spruce Street, Philadelphia, PA 19104 USA

## Abstract

**Background:**

In addition to HLA genetic incompatibility, non-HLA difference between donor and recipients of transplantation leading to allograft rejection are now becoming evident. We aimed to create a unique genome-wide platform to facilitate genomic research studies in transplant-related studies. We designed a genome-wide genotyping tool based on the most recent human genomic reference datasets, and included customization for known and potentially relevant metabolic and pharmacological loci relevant to transplantation.

**Methods:**

We describe here the design and implementation of a customized genome-wide genotyping array, the ‘TxArray’, comprising approximately 782,000 markers with tailored content for deeper capture of variants across *HLA*, *KIR*, pharmacogenomic, and metabolic loci important in transplantation. To test concordance and genotyping quality, we genotyped 85 HapMap samples on the array, including eight trios.

**Results:**

We show low Mendelian error rates and high concordance rates for HapMap samples (average parent-parent-child heritability of 0.997, and concordance of 0.996). We performed genotype imputation across autosomal regions, masking directly genotyped SNPs to assess imputation accuracy and report an accuracy of >0.962 for directly genotyped SNPs. We demonstrate much higher capture of the natural killer cell immunoglobulin-like receptor (*KIR*) region versus comparable platforms. Overall, we show that the genotyping quality and coverage of the TxArray is very high when compared to reference samples and to other genome-wide genotyping platforms.

**Conclusions:**

We have designed a comprehensive genome-wide genotyping tool which enables accurate association testing and imputation of ungenotyped SNPs, facilitating powerful and cost-effective large-scale genotyping of transplant-related studies.

**Electronic supplementary material:**

The online version of this article (doi:10.1186/s13073-015-0211-x) contains supplementary material, which is available to authorized users.

## Background

Since the Organ Procurement and Transplantation Network (OPTN) began its registry in 1987 until mid-2014 over 575,000 solid organ transplantations have been performed in the United States [1]. Although there have been considerable improvements in patient treatment pre- and post-transplant surgery and immunosuppressant therapies (IST), various grades of rejection are observed in up to 40 % of transplanted individuals within the first year post transplant [2], and affects approximately 60 % of transplanted individuals over the course of the graft lifetime thereby representing a major risk factor for graft damage and eventual graft loss [3, 4]. There are also significant risks post transplant ranging from severe adverse events to side-effects of ISTs including nephrotoxicity, hyperlipidemia, and new onset of diabetes after transplantation (NODAT) [5, 6].

Recent advances in genomic technologies and large-scale human reference maps such as the International HapMap Project have led to the development of genome-wide association studies (GWAS) utilizing cost-effective arrays that allow for the rapid interrogation of several hundreds of thousands single nucleotide polymorphisms (SNPs) and copy number variants (CNV) across the human genome [7–9]. Large scale whole genome sequencing studies show that approximately 3.5 million and approximately 10 million common and rare polymorphisms are typically observed between two unrelated individuals of European and African ancestries, respectively [10]. Each donor-recipient (D-R) pair of genomes contains vast permutations of non-synonymous amino-acid differences and other potential sources for allogenicity, beyond the highly characterized human leukocyte antigen (*HLA*) region, conventionally considered to contain the main genetic factors underpinning allograft rejection. Rejection is observed in following *HLA*-matched transplantations between full sibling, suggesting that histocompatibility may depend on non-*HLA* genetic differences. This includes a number of minor histocompatibility antigens, such as the H-Y antigens [11], which have been studied in the context of renal transplantation. Such findings from these studies identifying non-HLA histocompatibility loci suggest that non-*HLA* genetic disparities exist between D-Rs, and that these differences may manifest as the presentation of polymorphic peptides that the recipient’s immune system recognizes as non-self even in the presence of IST. Indeed, analyses of overall 10-year kidney graft failure rates for cadaver donors showed that 18 % of graft failures were due to *HLA* factors, as observed through mismatched living donor grafts; and 43 % were attributable to non-immunological factors, and 38 % of the failures were due to immunological reactions against non-HLA factors as seen in *HLA*-identical sibling grafts [4]. The natural killer cell immunoglobulin-like receptor (*KIR*) region comprising a family of 13 genes on chr19q13.46 are known to interact with HLA Class I molecules, and many unique *KIR* haplotypes identified have been linked to transplantation outcomes [12, 13] Additional non-*HLA/KIR* polymorphisms have also been shown to impact transplantation outcomes since through the generation histo-incompatibilities [14–16]. Investigations of non-*HLA* genetic determinants of clinical outcomes following organ transplantation have yet to be performed in any systematic well powered fashion to date.

A recent genome-wide study of NODAT was conducted in a prospective cohort of 529 kidney transplant recipients, 57 of whom developed NODAT with 26 SNPs identified in the discovery stage (*P* <1 × 10^−5^), eight of which retained association on replication, of which seven intriguingly are in loci known to have a role in Beta-cell apoptosis [17]. A number of genetic variants impacting uptake, metabolism, and excretion of immunosuppressant drugs have been identified [18]. While there are examples of robust associations in a number of these studies, validation of a large number of other putative associations in independent studies are often not observed [19]. This is likely to contribute to publication bias, underpowered discovery cohorts, and failure to adjust for population stratification.

The use of current sequencing and dense genotyping data from reference populations also makes it feasible to further infer, or impute, tens of millions of additional genotypes, which were not directly genotyped on the initial platform [20–22], by the use of whole genome imputation using highly characterized genomic reference datasets such as the 1000 genomes project (1KGP) and the Genomes of the Netherlands (GoNL) [23, 24]. Array-based genotyping technologies that have enabled conventional GWAS analyses also permit flexibility in choosing the scope and density of SNPs for disease or trait-specific arrays geared toward particular research communities. Such arrays include platforms such as the ‘cardiochip’ [25] and more recently the Immunochip and Cardio-Metabochip arrays [26, 27] have unveiled hundreds of new genetic associations leading to deeper understanding of the genetic architecture of new regions underpinning biological and disease processes. These newest arrays, including the Axiom Biobank and the UK Biobank genotyping arrays enable more comprehensive capture of genetic diversity across populations [28].

To create a unique genome-wide platform to facilitate genomic research studies in transplant-related studies, we designed a genome-wide genotyping tool customized for known and potentially relevant loci in metabolic and pharmacological aspects of transplantation including content relevant for D-R genomic incompatibility. We describe here the design and implementation of a genome-wide 782,000 marker array herein termed the ‘TxArray’ with tailored deeper capture of variants in *HLA*, *KIR*, pharmacogenomic, and metabolic genes/loci important in transplantation while still allowing conventional hypothesis-free GWAS to be performed. The genome-wide coverage of this array was created using content from conventional GWAS arrays [29, 30] with transplant-specific content informed from a range of sources including comprehensive literature searching and by expert opinions on priority pharmacogenomic loci. Our targeted customized modules are also designed to provide improved coverage of functional variations based on updated content from the 1KGP [10] and powerful analyses of over 32,000 exomes [31, 32].

DNA from over 16,000 DNA samples has thus far been genotyped using this array allowing for more robustly powered *in silico* replication as well analyses of rare variants and loss of function (LoF) variants ablating all or parts or a given gene, and cross-cohort meta-analyses in diverse populations. The majority of these samples are contributed as a part of International Genetics & Translational Research in Transplantation Network (iGeneTRAiN), a major international collaboration on the genomics of transplantation [33]. The objectives of forming this consortium are: (1) to pool expertise for selection of genes and SNPs; (2) to reduce costs by producing a standardized genome-wide genotyping platform; (3) to facilitate ease of cross cohort meta-analyses and replication for a large set of SNPs in high priority candidate genes; and (4) to bolster statistical power by combining as many of these appropriately harmonized datasets to discover new genes involved in a range of phenotypes and outcomes relating to solid organ and hematopoietic stem cell transplantation (HCT). Here we formally describe the rationale, design and content of our transplant genotyping array, and describe the imputation process as well as evaluate its performance in capturing variation across major populations.

## Methods

### Affymetrix genotyping platform and assay technology

The Axiom genotyping platform utilizes a two-color, ligation-based assay using 30-mer Oligonucleotide probes synthesized *in situ* onto a microarray substrate. There are approximately 1.38 million features available for experimental content with each feature approximately 3 μm^2^ with each SNP feature contains a unique oligonucleotide sequence complementary to the sequence flanking the polymorphic site on either the forward or the reverse strand. Solution probes bearing attachment sites for one of two dyes, depending on the 3' (SNP-site) base (A or T, versus C or G) are hybridized to the target complex, followed by ligation for specificity.

### Array design and variant selection

The transplant-specific modules and genome-wide content for the TxArray was designed based on a tiered system built on the main Affymetrix GWAS imputation grids [30] for the major human populations as defined by the Hapmap Project [9] and subsequent high density population reference studies yielding high density genomic datasets including representative individuals of European ancestry (Utah residents with ancestry from Northern and Western Europe (CEU)), of Asian descent (Japanese from Tokyo, Japan (JPT)), and Han Chinese from Beijing, China (CHB)), and of African ancestry (Yoruba in Ibadan, Nigeria (YRI)) and Americans of African Ancestry in SouthWest, USA (ASW)).

In addition to this core content, additional modules of SNPs were added sequentially so that maximal economy of markers was retained by ensuring no redundant SNPs were added. We describe the tiers sequentially below:

#### A. Cross-platform ‘cosmopolitan’ genome-wide coverage markers (approximately 350,000 markers)

- ***Genome-wide imputation grid*** (approximately 296K markers): The TxArray’s core imputation grid consists of genome-wide approximately 296K SNPs shared in common with the conventional Affymetrix Biobank Array. These include a set of 246K SNPs, also included in the UK Biobank array, that provide high-density coverage (mean r^2^ >0.81 and 0.90) across European populations (CEU) at minor allele frequencies (MAFs) >1 % and 5 %, respectively.
- ***Additional coverage for non-European populations:*** An additional set of approximately 50K SNPs, covered in the 1KGP Phase I reference panel, were additionally extracted from the Affymetrix-Biobank array to improve the mean coverage achieved in African and other populations. These SNPs were chosen with the goal of achieving comprehensive overlap with already existing UK Biobank Axiom Array and the Axiom Biobank Array, to facilitate additional collaborative efforts where joint or meta-analyses of samples genotyped across these platforms and other conventional GWAS platforms are required [29, 30].
- ***Compatibility markers*** (approximately 18K markers): This module was designed to optimize and standardize genotyping quality control (QC) and sample validation through the use of: Polymorphisms capturing Ancestry informative markers (AIMs); fingerprinting panels; mitochondrial, *Y-*chromosome; and miRNA binding sites or targets regions were included.

#### B. Module-specific content from the UK Biobank core array (approximately 36K markers)

**Fig. 1:**
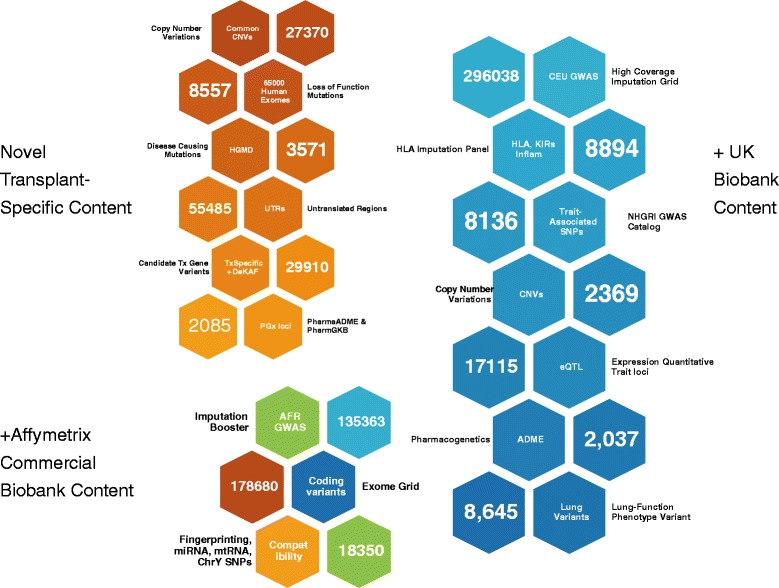
Key content modules included in the TxArray. Marker modules are divided into those in common with the UK Biobank Axiom Array, Axiom Biobank Genotyping Array, and those unique to the TxArray along with module descriptions and counts of unique markers in each category. The goal of preserving the specific markers already covered on the two existing biobank arrays is maximize shared coverage for future cross-study joint and meta-analyses that may utilize these popular state-of-the art genotyping platforms

These constitute markers identified based on reported GWAS signals and candidate gene associations across pharmacogenomic and metabolic phenotypes. Again, to enable cross-platform analysis, where feasible, we also included markers directly overlapping the UK-Biobank array and additional markers for the transplant-specific content. The following UK-Biobank array modules were included; see Fig. 1 and the UK-Biobank Consortium for details of included variants [29]:

1. *HLA* and *KIR* region markers (7,348 and 1,546 variants, respectively)
2. Known phenotype associations curated by the National Human Genome Research Institute (NHGRI) GWAS Catalog [34, 35], (8,136 variants)
3. Known CNVs (2,369 variants)
4. Expression-quantitative trail loci, or eQTLs (17,115 variants)
5. Lung-tissue specific or pulmonary function-associated markers (8,645 variants)

### Targeted *MHC* and transplant-specific modules

**Fig. 2:**
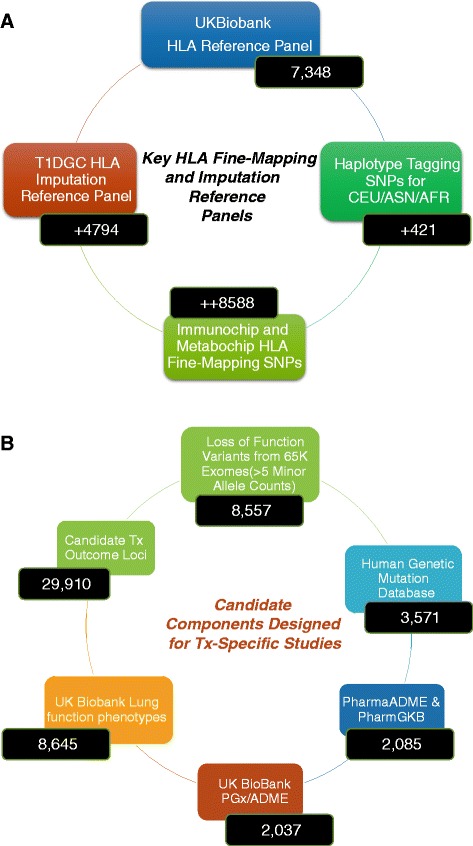
Modular content in the TxArray targeting the MHC and custom-designed for transplant-focused association studies. **a** MHC modules included in the TxArray and the total count of markers either directly tiled or tagged. Note that the marker counts are not unique as some modules overlap. A total of 10,820 and 13,428 unique markers across the *MHC* and extended *MHC* are included in the TxArray. **b** Major categories of transplant-specific markers included in the array. See methods and supplemental tables for details of design and content considerations

Specific modular content incorporated in the array dedicated to address transplant community research goals. Aside from the above-described modules overlapping with the UK-Biobank array, we expanded modules dedicated to non-*HLA* MHC region markers, deep coverage of known and predicted LoF variants, and untranslated regions (UTR)-specific module. Note that all positions and variants referenced herein are based on the human genome builds *hg19/build37* (Fig. 2).

### *MHC* and *KIR* content for fine-mapping and imputation

The TxArray provides the most current and densest coverage of the extended *MHC* (Chr 6:25.5MB to 34MB *hg19/build37*) [36, 37]. While the UK-Biobank array includes dense *HLA*-specific coverage, a number of *MHC* genes and markers mapping variants outside of the *HLA*-encoding regions are critical players in immune function and some have known roles in histocompatibility (for example, *MICA*, *MICB*). Thus, we included a comprehensive set of *MHC* markers in addition to the conventional *HLA*-coding regions (Fig. 2a).

Additionally, given the important role of *KIR* in allo-recognition through its interaction with *HLA*, we included additional *KIR* SNPs to enable fine-mapping, imputation, and structural variation association analysis, as well as interaction analyses across *KIR* and *HLA Class I*, which has a known role in histocompatibility in HCT, as well as other *MHC* loci.

To build this content and attempt to preserve significant overlap with state-of-the-art, popular genotyping platforms, we curated and included in our design content from the following resources and platforms (Fig. 2a and Additional file 1: Table S1):

1. UK-Biobank array (8,894 total variants), including 7,348 *HLA* markers and 1,546 KIR markers.
2. Multiethnic *HLA* haplotype tagging SNPs [36] (421 SNPs).
3. The Type 1 Diabetes Genetic Consortium (T1DGC) Imputation panel (4,794 SNPs included directly tiling or tagging by LD those SNPs in the *HLA* imputation panel for SNP2*HLA* [38]).
4. Non-redundant MHC validated SNPs from existing genotyping platforms used in large-scale studies: (1) Metabochip (1,123 SNPs) and (2) Immunochip (12,609 variants) [26, 27].

The content above includes 10,820 non-redundant SNP markers. We maximized the coverage of this content using a non-redundant set of best-tagging variants to achieve satisfactory tagging of the major HapMap continental populations including African (ASW and YRI), European (CEU), and Asian (CHD, JPT, CHB).

#### C. Transplant-specific content

##### Pharmacogenomic

Drug absorption, metabolism, excretion and toxicity markers, n = approximately 7,500 SNPs including markers derived from PharmGKB [39]. As these SNPs were of key relevance to this array, we also included at least one or more tagging SNPs to cover those common variants present in the 1KGP database. Literature searching was also performed (see below) for serious adverse events and pharmacogenomics studies related to IST and other therapeutics relating to transplantation. Previous candidate gene/pathway genotyping results from the Deterioration of Kidney Allograft Function (DeKAF) study were also included (n = approximately 2,000 SNPs) [40–42].

##### Candidate genes associated with transplant outcomes

Over 600 transplantation-related genetic association studies were manually curated from PubMed using the following search string: ‘transplant + DNA + donor + recipient AND (liver OR hepatic OR lung OR pulmonary OR heart OR cardiac OR kidney OR renal) AND (SNP OR polymorphism OR variant)’. Key information including PMID number, size and population examined, loci and SNPs studied (including the respective rsID numbers), and number of donors and recipient subjects were collated. An emphasis was placed on sample size, data quality and strength of the described associations to facilitate more powerful meta-analyses with data from existing publications.

To maximize the coverage in the CEU, YRI, and ASN populations, we selected an additional non-redundant set of 23.8K variants to boost coverage the total of 91.9K polymorphic sites included in these loci. The SNPs were chosen based on an algorithm that attempts to maximize the expected mean coverage across all three key populations simultaneously instead of one at a time. This was performed by selecting the tagging SNP marker that tags most SNP markers from all three populations first; this strategy enables identification of minimal SNP sets for maximal cross-ethnic coverage (see Additional file 1: Table S2).

In our comprehensive literature search we identified primary research and review articles across each of the major solid transplant organs, including heart, liver, kidney, lung, among others as well as hematopoietic stem cell transplantation. In addition to considering measurements of graft survival and all cause, or organ failure related mortality, we also considered genes previously implicated in transplant associated complications, such as new-onset diabetes after transplantation (NODAT) and response to transplant-related medications.

We included those previously identified gene candidates and wherever no specific candidate gene has been fine-mapped or independent replicated or validated, we mapped known SNP associations to nearby coding loci and included tagging variants and variants in LD to boost local coverage. We considered a number of recent studies that attempted to replicate GWAS findings [17, 19, 43], as well as a number of recent reviewers [44–47].

Full details of all the polymorphisms on the array including their chromosome position and additional annotations are outlined in [33].

#### Functional variants modules

Aside from the modules noted as being shared with the UK-Biobank, the following categories of variants were included in the design for this component:

- ***Affymetrix Biobank array content:*** We considered a total of approximately 250,000 SNPs from the Axiom Biobank Genotyping Array, including 86,000 putative exonic SNVs and putative LoF variants. As not all of these have been validated and many are not polymorphic in the general populations, we used one of the largest whole-exome sequencing reference datasets available at the time of the design, comprising over 32,000 samples, to annotate and filter these variants based on the observed minor allele counts (MACs). We included only those variants with MACs greater than five observations in this database, which yielded approximately 168K exonic or coding variants and over 16K putative LoF variants. A total of 178,680 unique variants were selected in this module.
- ***Human Gene Mutation Database (HGMD):*** We curated variants of The HGMD LoF database (up until 1 August 2013). Again, as above, we only included MAC observed greater than five times, for a total of 3,571 variants (See Additional file 1: Table S3).
- ***Additional LoF variants*:** Using the above-noted approximately 32,000 exome database, we identified additional putative LoFs included in the Affymetrix Biobank Genotyping Array, UK Biobank Axiom Array, or HGMD databases; again, filtering the observed SNVs and indels from analysis across over 32,000 human exomes [32] for at least MACs greater than 5, we obtained a conservative set of 8,557 unique putative exonic SNVs and/or putative LoF variants (See Additional file 1: Table S3).
- ***Untranslated Region (UTR) Coverage:*** To provide maximal coverage of SNPs that may affect functional gene expression, we additionally focused on the coverage of 5’ (and 3’) UTRs defined as the exonic region between the transcriptional start (stop) and translational start (stop) sites as defined by either the RefSeq or ENSEMBLE human genome (hg19) reference sequences in June 2013. Using a MAF cutoff for inclusion of >1 % or 5 % in CEU and AFR (ASW + YRI) populations, respectively, we included a total of approximately 184,000 SNPs as shown in Additional file 1: Table S4 and described in the Supplementary Material.
- ***A priori associations:*** To focus on known phenotypes, 8,136 SNPs that reached a conventional GWS threshold at *P* <5 × 10^−8^ (December 2012) for both quantitative traits and disease-specific reported in NHGRI GWAS Catalog were included.

### Copy number variations (CNVs) and polymorphisms (CNPs)

#### CNP tagging and regional coverage

To cover common genomic structural elements by SNP-tagging we included 5,410 markers (See Additional file 1: Table S5A) and we used an additional 21,960 variants to cover approximately 2,200 manually curated CNV regions as described in the Supplementary Materials.

### E. GWAS booster

The GWAS ‘booster module’ includes a set of additional markers (on top of the markers included in the main modules) selected by identifying the minimal set of markers that will provide the optimal added coverage value (with regard to the best overall coverage for whole genome imputation). Since the goal is to fill the array and gain additional coverage with the minimal number of markers, we used LD-based pair-wise tagging. Specifically, we focused on improving the coverage of common variants (MAF >2 % in CEU and MAF >5 % in AFR populations) by selectively adding additional variants resulting in selection a total of 135,363 additional markers based on the projected improvements in the overall coverage (Additional file 2: Figure S1). The online resource [33] outlines a comprehensive list of SNPs and genes for the TxArray.

This study conformed to the Helsinki Declaration as well as to local legislation. Informed and written consent was obtained independently for each iGeneTRAIN study participant, with appropriate oversight and approvals from respective local institutional review boards/Research Ethics Committees to use either summary-level or anonymized individual-level data. A number of our GWA studies are mandated to release their datasets into dbGAP under their funding conditions and subject to the ethical consents in place. We will update these dbGAP uploads on the [33] site every 3 months.

## Results

### Quality control

Assays for approximately 782,000 markers were manufactured following recommendations based on the Affymetrix Best Practices protocol [48] for performing genotype marker QC using a merged set of 4,885 DNA samples including those from the DeKAF study site and samples genotyped expressly for the purpose of quality control assessment. The latter consists of 85 samples from the HapMap project. Genotype clustering efficiency was also performed per manufacturer’s recommendations based on unique parameters established for this specific custom-design array, which consists of a high number of markers covering loss of function and copy number variable regions that may not be polymorphic in the vast majority of the population.

### Genotype concordance with HapMap, 1KGP panels, and duplicate samples

We genotyped 85 HapMap samples on the TxArray to test concordance and genotyping quality. We included 48, 24, and 13 samples of European (CEU), Asian (JPT, CHB), and African (YRI) ancestry, respectively. All analyses were performed with PLINK (1.07/1.9) [49]. First, we examined eight trios (four CEU, four YRI) for Mendelian errors. All 767,203 genotyped SNPs passing QC based on manufacturer’s metrics were included and Mendelian inconsistency was calculated based on the number of total instances where a child’s genotypes at a given SNP position are not attributable to that of either parent, for instance if both parents have an AA genotype, while the child has AB. The number of SNPs errors for each of the eight families varied between 264 and 4,672, which corresponds to a parent-parent-child (P-P-C) heritability greater than 0.993 and an average of 0.997 (Table 1).

**Table 1:** Mendelian consistencies among HapMap family samples genotyped

Ancestry	FID	HapMap father	HapMap mother	HapMap child	SNP errors observed	P-P-C heritability* (%)
CEU	1334	NA12144	NA12145	NA10846	3,757	99.51
CEU	1340	NA06994	NA07000	NA07029	3,600	99.53
CEU	1340	NA07022	NA07056	NA07019	264	99.97
CEU	1463	NA12891	NA12892	NA12878	869	99.89
YRI	Y004	NA18501	NA18502	NA18500	4,397	99.43
YRI	Y009	NA18507	NA18508	NA18506	4,672	99.39
YRI	Y045	NA19200	NA19201	NA19202	602	99.92
YRI	Y058	NA19223	NA19222	NA19221	936	99.88

*Heritability calculated as (# correct /767203 total SNPs post-QC)

CEU, International HapMap CEPH European descent populations; FID, HapMap family ID; P-P-C, parent-parent-child; YRI, Hapmap Yoruba African samples

Next, we investigated concordance between 279,061 genotypes overlapping between our genotyping array and HapMap2 (r22, b36). We tested 22,944,075 sample-SNP combinations for concordance and observed a concordance rate of 0.996. Concordance rates for the three populations (African, Asian, and European) were very similar (Table 2). As our array is specifically set up to test MHC and X chromosome SNPs, we also tested SNPs in these two regions. Results are comparable to the overall concordance: The concordance rate for the *MHC* SNPs is 0.994, and 0.998 for SNPs on the X chromosome (Table 2). We also performed this analysis using data from the 1KGP reference panel and observed comparably high concordance rates (Table 2). Overall, we show that the genotyping quality of the TxArray is high, which enables accurate association testing and imputation of ungenotyped SNPs using reference panels such as 1KGP and Go-NL consortia.

**Table 2:** Genotyping concordance rates across HapMap and 1000 Genomes Panel samples genotyped on the TxArray

SNPs	Reference panel	Ancestry	Number of samples	Total overlapping SNPs post-QC*	Total SNPs concordant	Concordance (%)
All	HM2	ALL	85	22,944,075	22,843,908	99.56
All	HM2	EUR	48	13,058,670	12,986,021	99.44
All	HM2	ASN	24	6,414,064	6,396,803	99.73
All	HM2	AFR	13	3,471,341	3,461,084	99.70
Chr X	HM2	ALL	85	515,791	514,641	99.78
MHC**	HM2	ALL	85	619,108	615,244	99.38
All	1KGP	ALL	55	36,430,433	36,241,576	99.48
All	1KGP	EUR	27	17,830,297	17,716,762	99.36
All	1KGP	ASN	21	13,953,847	13,905,332	99.65
All	1KGP	AFR	7	4,646,289	4,619,482	99.42
Chr X	1KGP	ALL	55	937,853	933,799	99.57
MHC**	1KGP	ALL	55	232,410	230,458	99.16

*Total number of SNPs tested such that each SNP that is non-missing for all samples is counted 85 times

**Defined as between chr6:25.5Mb and chr6:34Mb (hg19)

HM2, International HapMap 2 dataset; 1KGP, 1000 Genomes Project reference dataset

Fifty duplicate pairs across 12 cohorts were genotyped using the TxArray. To assess the quality of the genotyping array, we tested concordance of all SNPs that were non-missing in both samples. In assessments of between approximately 742,000 and 765,000 SNPs we observed that on average 99.657 % of SNPs were fully concordant (that is, both alleles correspond), while 0.341 % of SNPs had a single concordant allele and only 0.002 % of SNPs were fully discordant.

### Comparisons with conventional GWAS platforms

#### Coverage of the 1KGP panel markers

We compared the mean coverage (based on maximum achievable r2) across common markers (either MAF >0.05 or 0.01) in the 1KGP achieved by either the marker content designed on the TxArray versus that by a number of conventional genotyping platforms (that is, Infinium 1M and 660K Beadchips (Illumina), and Genome-Wide Human SNP Array 6.0 (Affymetrix)).

**Fig. 3:**
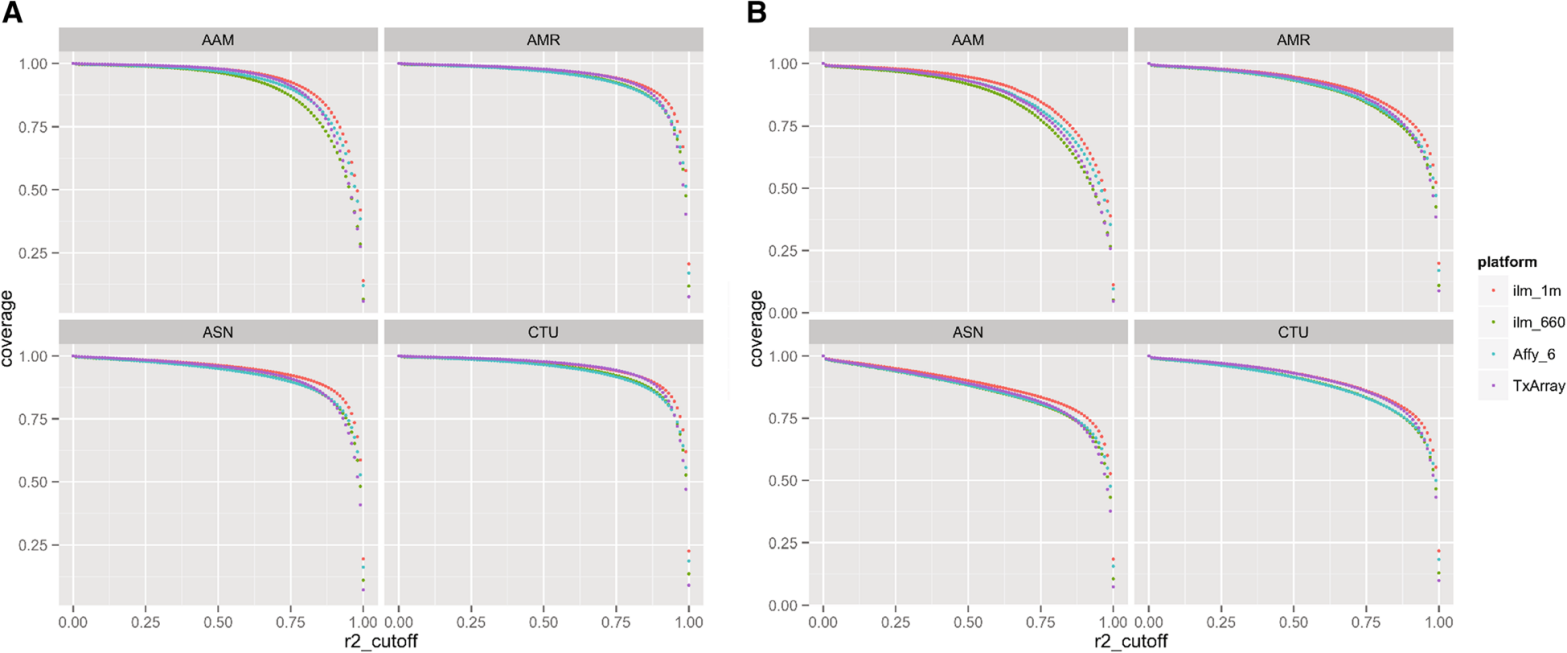
Comparison of coverage of 1000 genomes project reference panel between TxArray and other genome-wide genotyping platforms for variants with MAF >0.05 (**a**) and >0.01 (**b**). Coverage (ordinate) for the 1000 Genomes Project Phase I integrated reference panel was assessed using maximum *r* ^*2*^ (abscissa), at an MAF cutoff of 0.05 (**a**) and 0.01 (**b**). Populations included: (1) African ancestry (AAM): Yoruba in Ibadan, Nigeria (YRI) and Americans of African Ancestry in SouthWest, USA (ASW); (2) Admixed American (AMR): Colombians from Medellin, Colombia (CLM), Mexican Ancestry from Los Angeles USA (MXL), and Puerto Ricans from Puerto Rico (PUR); (3) Asian (ASN): Han Chinese in Beijing (CHB), Southern Han Chinese (CHS), Japanese in Tokyo, Japan (JPT); and (4) European ancestry (CTI): Utah residents with ancestry from Northern Western Europe (CTU), Central and Eastern European (CEU), and Toscani in Italia (TSI), as described in the HapMap and 1KGP. The platforms compared include the TxArray using 767,203 SNPs passing QC. ILMN_1M and ILMN660 refer to Illumina’s Infinium one million and the Illumina 660K genotyping platforms. Affy_6.0 refers to the Affymetrix 6.0 SNP chip containing approximately 906,600 SNPs

Figure 3a and b shows the composite coverage of markers in the 1KGP panel by the markers genotyped on the TxArray versus other conventional GWAS products for European (CEU and Toscani in Italia (TSI)), African (ASW/YRI), Admixed American (AMR) (Colombians from Medellin, Colombia (CLM), Mexican Ancestry from Los Angeles USA (MXL), and Puerto Ricans from Puerto Rico (PUR)), and Asian (ASN) (CHB, Southern Han Chinese (CHS), and JPT) individuals using MAF cutoffs of >0.01 and >0.05 for the full range of r^2^ cutoff thresholds (from r^2^ = 0 to 1). The TxArray performed comparably next to these other genotyping SNP chips, which were designed to provide optimal genome-wide coverage even though the TxArray devoted a significant number of markers to transplant-specific, rare loss of function and *MHC/KIR* specific content.

#### Coverage of exonic, MHC, and KIR locus markers

**Fig. 4:**
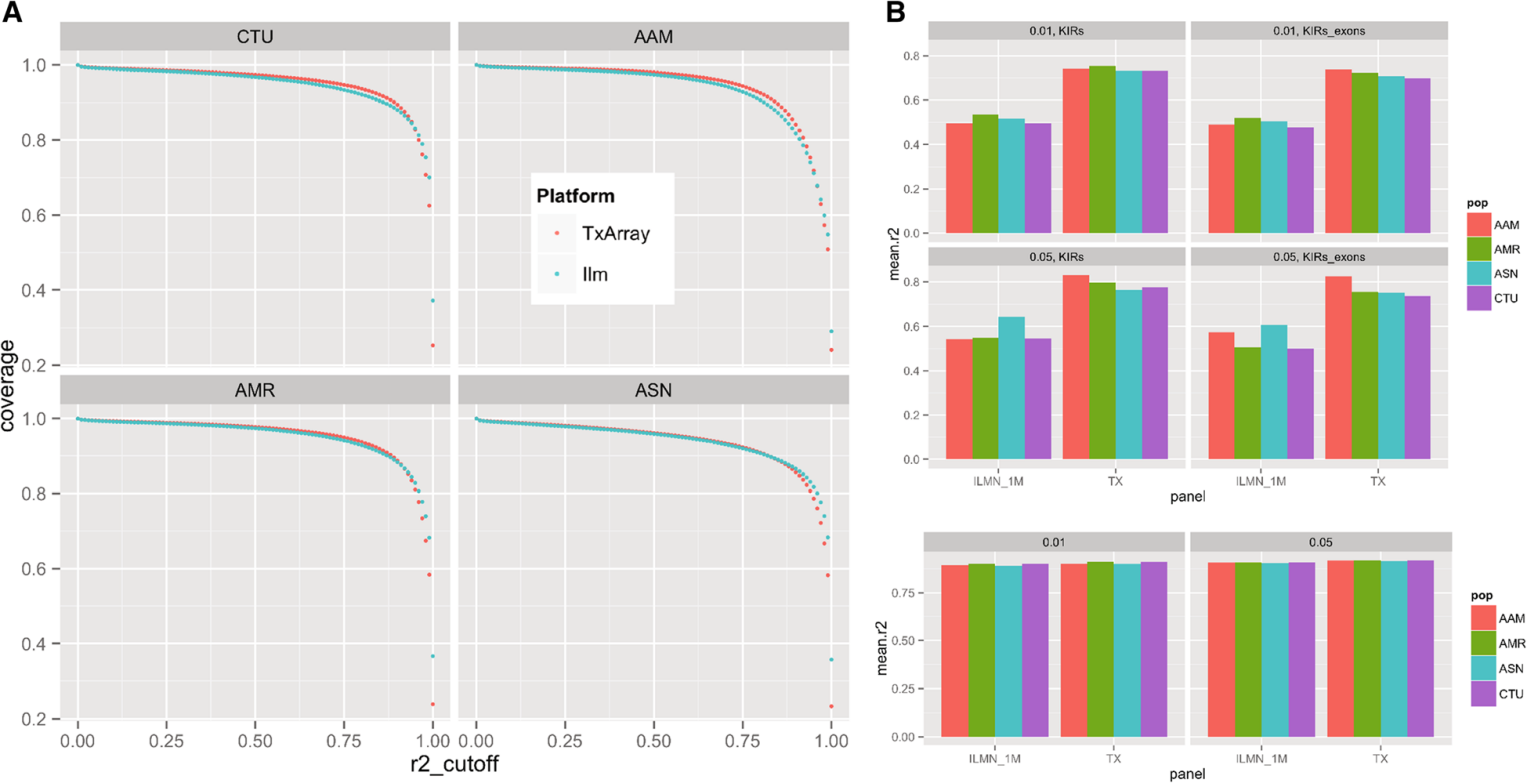
Comparison of coverage between TxArray and ILMN_1M genotyping platforms across exonic regions, the extended MHC and the KIR-encoding locus. **a** Coverage (ordinate) for all exonic markers and UTR region markers in the 1000 genomes reference panel was assessed using max r2 (abscissa), at an MAF cutoff of 0.05 (a) and 0.01 (b), in (1) European ancestry ((CEU) and Tuscany in Italia (TSI)); (2) African ancestry (AAM) (Yoruba in Ibadan, Nigeria (YRI)) and Americans of African Ancestry in SouthWest, USA (ASW); (3) Admixed American (AMR) (Colombians from Medellin, Colombia (CLM), Mexican Ancestry from Los Angeles, USA (MXL), and Puerto Ricans from Puerto Rico (PUR)); and (4) Asian (ASN) (Han Chinese in Beijing (CHB), Southern Han Chinese (CHS), Japanese in Tokyo, Japan (JPT)) HapMap and 1KGP individuals. The compared platforms include the TxArray using 767,203 SNPs that passed manufacturing and standard genotyping QC. ILMN_1M refer to Illumina’s Infinium one million SNP GWAS array. **b** Comparison of coverage across variants within *KIR*-encoding regions using the TxArray (TX) or the Illumina 1M (ILMN_1M) genotyping platforms across the four major HapMap populations (European (CTU): CEU+TSI; AAM: ASW+YRI; AMR: CLM+MXL+PUR; ASN: CHB+CHS+JPT). Coverage is based on mean r^2^ of variants included in the 1000 genomes phase I reference panel with a MAF of >0.01 (top) or >0.05 (bottom). *KIR* genes included: (*KIR2DP1*, *KIR2DL1*, *KIR2DL2*, *KIR2DL3*, *KIR2DL4*, *KIR2DL5A*, *KIR2DL5B*, *KIR2DS1*, *KIR2DS2*, *KIR2DS3*, *KIR2DS4*, *KIR2DS5*, *KIR3DL1*, *KIR3DL2*, *KIR3DL3*, *KIR3DP1*, *KIR3DS1*, *KIR3DX1*). Coverage was compared for either all *KIR* region markers (left) or only those in exonic regions (right). **c** Comparison of coverage across the extended *MHC* (25,500,000–34,000,000) using either the TxArray (TX) or the Illumina 1M (ILMN_1M) genotyping platforms across the four major HAPMAP populations (CTU: CEU/TSI; AAM: ASW/YRI; AMR: CLM/MXL/PUR; ASN: CHB/CHS/JPT). Coverage rate is calculated based on the mean achieved r^2^ for variants included in the 1000 Genomes Project (1KGP) Phase I reference panel with a MAF of >0.01 (left) or >0.05 (right)

The TxArray also provided efficient coverage of markers across the exonic, *KIR*, and *MHC* regions when compared to the commonly-used Illumina 1M platform (Fig. 4a, b, and c, respectively). While mean expected coverage is comparable for the exonic and *MHC* regions, the TxArray provides a significantly improved coverage of markers across the *KIR* locus, which has been a region that has arguably received insufficient attention in most transplant association studies.

### Imputation

We performed genotype imputation across all autosomal regions for 12 iGeneTRAIN studies (n = 12,048 post-QC GWAS samples) using ShapeIT2/ IMPUTE2 with the 1KGP reference panel (v3), resulting in approximately 38 million variant calls. We masked 0.2 % directly genotyped SNPs, with separate imputation performed with and without these 0.2 % SNPs to assess proxy genome-wide imputation accuracy. We report an accuracy in the range of 96.24 % to 97.71 % for directly genotyped SNPs across the genome for the 12,048 GWAS datasets imputed.

We looked at number of SNPs per MAF bins (0.01 intervals) in all imputed data. We observed that INFO score (quality metric to estimate uncertainty in imputation) for variants below MAF 0.05 declines but as MAF increases, INFO score also increases. In most cases, all variants above MAF 0.05 have INFO scores greater than 0.8 (data not shown). From masked analysis, we looked at concordance among each masked SNP where INFO scores were greater than 0.8, and results indicated very high concordance among all masked SNPs. Comparison of imputation accuracy/metrics from two independent pipelines (both using ShapeIT/IMPUTE2 with 1KGP as the reference population) was performed for The Genomics of Chronic Renal Allograft Rejection (Go-CAR) Study at Penn State and Mount Sinai and >99.998 % of imputed SNPs were concordant.

We looked at number of SNPs per MAF bins (0.01 intervals) in all imputed data as well as after performing masked analysis where 0.1 % of genotyped markers were removed and imputed again to assess the accuracy of our imputation. We observed that INFO score (quality metric to estimate uncertainty in imputation) for variants below MAF 0.05 declines but as MAF increases, info score also increases. In most cases, all variants above MAF 0.05 have info score greater than 0.8.

Using Beagle version 3.0.4, we imputed classical alleles and amino acid polymorphisms in *HLA-A*, *HLA-B*, *HLA-C*, *HLA-DPA1*, *HLA-DPB1*, *HLA-DQA1*, *HLA-DQB1*, and *HLA-DRB1* at a four-digit resolution, as well as an additional 3,117 *MHC* SNPs. We used data collected by the T1DGC as a reference panel, which include 5,225 individuals of European descent. Methods have been described previously in more detail [38, 50].

## Discussion

We have designed and implemented a genome-wide SNP array tailored for deeper capture of variation in loci of high priority in transplantation. Our primary goal in the array design was to generate a low-cost genome-wide array while maximizing coverage of known or putative transplant-related content. In attempts to unveil allogenicity between D-R genomes we also augmented the custom content from all available resources for rarer LoF variants that may not be identified using traditional association studies based on imputation. Flexibility in SNP selection afforded the ability to: (1) ensure selective and consistent coverage for a range of prioritized loci across multiple ancestries; (2) provide deeper coverage beyond conventional HapMap populations; (3) to directly assay specific SNPs derived from previously published transplant studies; and (4) updated *HLA* and *KIR*, pharmacogenomic, and LoF variants. We demonstrate much deeper coverage in high priority regions such as *KIR* and tag SNPs in these regions provide much better coverage for populations of African ancestry relative to existing GWAS products.

With the recent reports of *bona fide* associations transplant outcomes and/or pharmacokinetics of immunosuppression medication with genetic polymorphisms in transplant-related genes (for example, *APOL1*, *IL28B*, *CYP3A4/5*) [51–54], one of the current challenges will be to determine how these variants and loci at a molecular and mechanistic level and how they interact with other variants with drugs used in therapy and prevention, towards intermediate and clinical phenotypes.

In designing the transplant SNP v1 array we have also targeted all nsSNPs >MAF 0.01 using information from both HapMap and 1KGP and have tagged to MAFs >0.02 for a large number of key loci related to key transplantation outcomes such as pharmacogenomic and metabolic-related traits. We have also updated the *HLA* and *KIR* content with the most-extensive content known to date. As with other recent custom genotyping arrays in cardiovascular diseases and metabolism as well as in autoimmune diseases, the TxArray facilitates conduct of powerful and cost-effective large-scale genotyping of transplant-related studies. Such platform enable integration with additional ‘omics’ datasets such as transcriptomics, proteomics, and metabolomics to provide richer analyses in a number of the studies using this tool. Additionally, as other transplant related cohorts are utilizing this TxArray for GWAS, validation of results and comprehensive meta-analyses will be much more robust. The TxArray achieves dramatic reduction compared to designing single-trait follow-up reagents, and provides the opportunity for transplant community researchers to perform unbiased genome-wide analysis and cross-consortium independent replications.

## Conclusions

We report the design and implementation of a state-of-the-art, powerful oligonucleotide array that is optimized to interrogate the genome for associations with transplant-related phenotypes and outcomes. This array, the TxArray, includes independent modules that encompass fine-mapping SNPs mapping across the MHC useful for HLA imputation, in drug-response associated loci for the study of pharmacogenomics and adverse drug response, previously-reported genes associated with transplant-related outcomes, among others, that are of high priority among the transplant community. With the advent of this array and the formation of the iGeneTRAIN consortium, it is our aim that the downstream application of such genomics technologies can ultimately generate associations which will be applied as personalized and precision-oriented genomic tools to solve clinical questions and improving patient outcomes in transplantation.

## Additional files

Additional file 1: Table S1. Tagging and coverage of MHC region markers. **Table S2:** Tagging and coverage of Tx-specific genes. **Table S3:** Untranslated regions (UTRs) considered in the TxArray design. **Table S4:** Loss-of-function variants included in the TxArray. **Table S5:** Copy number polymorphisms (CNPs) and variations (CNVs) included in the TxArray. (DOCX 54 kb) Additional file 2: Figure S1. TxArray transplant-specific modular contents. (PDF 140 kb)

## Abbreviations

1KGP: 1000 genome project
APOL1: Apolipoprotein L1
CNV: copy number variants
CYP3A: Cytochrome P450 3A
D-Rs: donor-recipient pairs
GoNL: Genomes of the Netherlands
GWAS: genome-wide association studies
HCT: hematopoietic cell transplantation
HGMD: Human Gene Mutation Database
IL28B: Interleukin 28B
ISTs: Immunosuppression therapies
KIR: natural killer cell immunoglobulin-like receptor
LoF: loss of function
MAFs: minor allele frequencies
MICA and MICB: MHC class I polypeptide-related sequence-A and –B
NODAT: new onset of diabetes after transplantation
OPTN: Organ Procurement and Transplantation Network
SAE: severe adverse events
SNPs: single nucleotide polymorphisms
T1DGC: Type 1 Diabetes Genetic Consortium
UTR: Untranslated Region
