# Supplementary material for: Concept and design of a genome-wide association genotyping array tailored for transplantation-specific studies
Source: Genome Med. 2015 Oct 1;7:90. doi: 10.1186/s13073-015-0211-x (PMC4589899; doi:10.1186/s13073-015-0211-x)
Supplement: Additional file 1: Table S1. — Tagging and coverage of MHC region markers. Table S2: Tagging and coverage of Tx-specific genes. Table S3: Untranslated regions (UTRs) considered in the TxArray design. Table S4: Loss-of-function variants included in the TxArray. Table S5: Copy number polymorphisms (CNPs) and variations (CNVs) included in the TxArray. (DOCX 54 kb) [file 13073_2015_211_MOESM1_ESM.docx]

**Supplementary Tables for “Concept and Design of a Genome-wide Association Genotyping Array Tailored for Transplantation-Specific Studies”**

Yun R Li^1,2^, Jessica van Setten^3^, Shefali S Verma ^6^, Yontao Lu^4^, Michael V Holmes^5^, Hui Gao^2,5^, Monkol Lek^7,8^, Nikhil Nair^2,5^, Hareesh Chandrupatla^2,5^, Baoli Chang^2,5^, Konrad J Karczewski ^7,8^, Chanel Wong^2,5^, Maede Mohebnasab^2^, Eyas Mukhtar^2,5^, Vinicius Tragante^3^, Cuiping Li^2^, Laura Steel^2,5^, Takesha Lee^2,5^, James Garifallou^2^, Toumy Guettouche^2^, Hongzhi Cao^10,11^, Weihua Guan^12^, Aubree Himes^2,5^, Jacob van Houten^2^, Andrew Pasquier^2^, Reina Yu^2^, Elena Carrigan^2^, Michael B Miller^13^, David Schladt^26^, Abdullah Akdere^1^, Ana Gonzalez^1^, Kelsey M Llyod^1^, Daniel McGinn^1^, Abhinav Gangasani^1^, Zach Michaud^1^, Abigail Colasacco^1^, James Snyder^2^, Kelly Thomas^2^, Tiancheng Wang^2^, Baolin Wu^12^, Alhusain J Alzahrani^25^, Amein K Al-Ali^14^, Fahad A Al-Muhanna ^14^, Abdullah M. Al-Rubaish^14^, Samir Al-Mueilo^14^, Dimitri S Monos^15, 2^, Barbara Murphy^16^, Kim M Olthoff^5^, Cisca Wijmenga^17^, Teresa Webster^4^, Malek Kamoun^15^, Suganthi Balasubramanian^18^, Matthew B Lanktree^5^, William S Oetting^19^, Pablo Garcia-Pavia^20^, Daniel G MacArthur ^7,8^, Paul IW de Bakker^9^, Hakon Hakonarson^2^, Kelly A Birdwell^21^, Pamala A Jacobson^22^, Marylyn D Ritchie^6^, Folkert W Asselbergs^3,23,24^, Ajay K Israni^27^, Abraham Shaked^5^, Brendan J Keating^5,28, 2 †.^

^1^ Medical Scientist Training Program, Perelman School of Medicine, University of Pennsylvania, Philadelphia, PA, USA

^2^ The Children’s Hospital of Philadelphia, Philadelphia, PA, USA

^3^ Department of Cardiology, Division of Heart and Lungs, University Medical Center Utrecht, Utrecht, The Netherlands.

^4^ Affymetrix Incorporated, Santa Clara, CA, USA

^5^ Penn Transplant Institute, Hospital of the University of Pennsylvania, Philadelphia, PA, USA

^6^ Center for Systems Genomics, The Pennsylvania State University, University Park, PA, USA

^7^ Analytic and Translational Genetics Unit, Massachusetts General Hospital, Boston, MA, USA

^8^ Program in Medical and Population Genetics, Broad Institute of Harvard and MIT, Cambridge, MA, USA

^9^ Department of Medical Genetics, Center for Molecular Medicine and Department of Epidemiology, Julius Center for Health Sciences and Primary Care, University Medical Center Utrecht, Utrecht, The Netherlands

^10^ BGI-Shenzhen, Shenzhen, China

^11^ Department of Biology, University of Copenhagen, Copenhagen, Denmark.

^12^ Division of Biostatistics, University of Minnesota, Minneapolis, MN, USA

^13^ University of Minnesota, Department of Psychology, Minneapolis, MN, USA

^14^ College of Medicine, University of Dammam, Kingdom of Saudi Arabia

^15^ Department of Pathology and Laboratory Medicine, Perelman School of Medicine, University of Pennsylvania and the Children’s Hospital of Philadelphia. PA, USA

^16^ Division of Nephrology and Department of Medicine, Icahn School of Medicine at Mount Sinai, New York, NY, USA.

^17^ Department of Genetics, The University Medical Center Groningen, Groningen, The Netherlands

^18^ Program in Computational Biology and Bioinformatics, and Molecular Biophysics and Biochemistry Department, Yale University, New Haven, CT 06520, USA.

^19^ Experimental and Clinical Pharmacology, University of Minnesota, Minneapolis, MN, USA

^20^ Heart Failure and Inherited Cardiac Diseases Unit, Department of Cardiology, Hospital Universitario Puerta de Hierro Majadahonda, Madrid, Spain

^21^ School of Medicine, Vanderbilt University, TN, USA

^22^ College of Pharmacy, University of Minnesota, USA

^23^ Durrer Center for Cardiogenetic Research, ICIN-Netherlands Heart Institute, Utrecht, The Netherlands

^24^ Institute of Cardiovascular Science, faculty of Population Health Sciences, University College London, London, United Kingdom

^25^ Department of Clinical Laboratories Sciences, College of Applied Medical Sciences, King Saud University, Riyadh, Saudi Arabia

^26^ Minneapolis Medical Research Foundation, Hennepin County Medical Center, Minneapolis, MN, USA

^27^ Hennepin County Medical Center, University of Minneosta, Minneapolis, MN, USA

^28^ Department of Pediatrics, University of Pennsylvania, PA, USA

† To whom correspondence should be addressed

Brendan J Keating D.Phil.

Division of Transplantation, 2 Dulles, Hospital of the University of Pennsylvania

3400 Spruce Street, Philadelphia, PA 19104

Tel: +1 (267) 7604507

Email: [bkeating@mail.med.upenn.edu](mailto:bkeating@mail.med.upenn.edu)

**SUPPLEMENTARY TABLES**

**Table S1: Tagging and Coverage of MHC Region Markers**

1. **Targeted *MHC/KIR* region SNPs**

|  | SNPs considered | Directly tiled | Tagging Target* |
| --- | --- | --- | --- |
| HLA multiethnic Haplotype tagging SNPs | 517 | 406 | 111 |
| T1DGC | 5654 | 4630 | 1024 |
| Immunochip | 12609 | NA | 12609 |
| Metabochip | 1123 | NA | 1123 |

*target markers used for pair-wise tagging

1. **Coverage achieved per population for targeted *MHC/KIR* region SNPs**

|  |  | HLA Haplotype tagger SNPs | Immunochip | Illumina 550 | metabochip | T1DGC |
| --- | --- | --- | --- | --- | --- | --- |
| Group | Total Considered | 517 | 12609 | 250 | 1123 | 5654 |
| EUR | Target | 94 | 9674 | 204 | 1061 | 975 |
|  | Covered | 77 | 9350 | 200 | 995 | 937 |
|  | Coverage | 0.819 | 0.967 | 0.98 | 0.938 | 0.961 |
| EAS | Target | 92 | 7600 | 191 | 1022 | 889 |
|  | Covered | 74 | 7407 | 187 | 954 | 854 |
|  | Coverage | 0.804 | 0.975 | 0.979 | 0.933 | 0.961 |
| ASW | Target | 96 | 10389 | 206 | 1092 | 984 |
|  | Covered | 78 | 10101 | 202 | 1025 | 946 |
|  | Coverage | 0.813 | 0.972 | 0.981 | 0.939 | 0.961 |
| YRI | Target | 93 | 9421 | 196 | 1033 | 929 |
|  | Covered | 75 | 9194 | 192 | 967 | 891 |
|  | Coverage | 0.806 | 0.976 | 0.98 | 0.936 | 0.959 |
| MEAN | Target | 93.75 | 9271 | 199 | 1052 | 944 |
|  | Covered | 76 | 9013 | 195 | 985 | 907 |
|  | Coverage | **81.05%** | **97.25%** | **98.00%** | **93.65%** | **96.05%** |

**Table S2: Tagging and Coverage of Tx-Specific Genes**

|  | EUR | ASW | YRI |
| --- | --- | --- | --- |
| Target (n) | 71,573 | 90,870 | 86,883 |
| Covered (n) | 62,658 | 75,600 | 71,907 |
| Coverage (proportion) | 0.88 | 0.83 | 0.83 |

**Table S3: Loss of Function variants included in the TxArray**

|  | HGMD | LoFs from analysis of 65K exomes |
| --- | --- | --- |
| Number of variants considered | 3697 | 8816 |
| Tiled by existing, validated SNPs | 2722 | 3945 |

**Table S4: Untranslated regions (UTRs) considered in the TxArray Design**

|  | EUR (>=1%) | ASW (>=5%) | YRI (>=5%) |
| --- | --- | --- | --- |
| Total variants considered | 130605 | 123175 | 125380 |
| Variants included or can be imputed from core grid | 86152 | 65044 | 67906 |
| Remaining target UTR variants considered | 44453 | 58131 | 57474 |
| Remaining target UTR variants tagged | 22356 | 41726 | 40926 |
| Mean coverage achieved | 0.831 | 0.867 | 0.868 |

**Table S5: Copy Number Polymorphisms (CNPs) and Variations (CNVs) included in the TxArray**

1. CNPs

| Variants considered | 6263 |
| --- | --- |
| Un-ambiguously mapped | 6258 |
| Tiled by SNPs validated by Axiom Assay | 4972 |
| Additional variants tagged | 1208 |
| Total markers included | 6180 |

1. CNVs

| *size of CNVs* | *# of CNVs* | *Median* | *Mean* | *Mean* | *Median* |
| --- | --- | --- | --- | --- | --- |
|  |  | ***# of markers*** | ***# of markers*** | ***size of gap*** | ***Size of gaps*** |
| (0,1000] | 38 | 3 | 3.026 | 194.7 | 196 |
| (1000,5000] | 273 | 9 | 9.696 | 315.5 | 333 |
| (5000,10000] | 288 | 21 | 21.06 | 368.4 | 364 |
| (10000,100000] | 921 | 23 | 26.96 | 1299.8 | 999 |
| >=100000 | 698 | 122 | 217 | 4895.9 | 2602 |

Abbreviations: EUR refers to HapMap and 1000 genome project (1KGP) European samples (Toscani in Italia; Finnish in Finland; British in England and Scotland; Iberian population in Spain); EAS refers to East Asian (Han Chinese in Beijing [CHB], Southern Han Chinese [CHS], Japanese in Tokyo, Japan [JPT]) HapMap and 1KGP populations; ASW and YRI refer to Americans of African Ancestry in SouthWest, USA and Yoruba in Ibadan, Nigeria respectively.
