# Supplementary figures and images for: Concept and design of a genome-wide association genotyping array tailored for transplantation-specific studies
Source: Genome Med. 2015 Oct 1;7:90. doi: 10.1186/s13073-015-0211-x (PMC4589899; doi:10.1186/s13073-015-0211-x)

Figure S1 TxArray Transplant-Specific Modular Contents

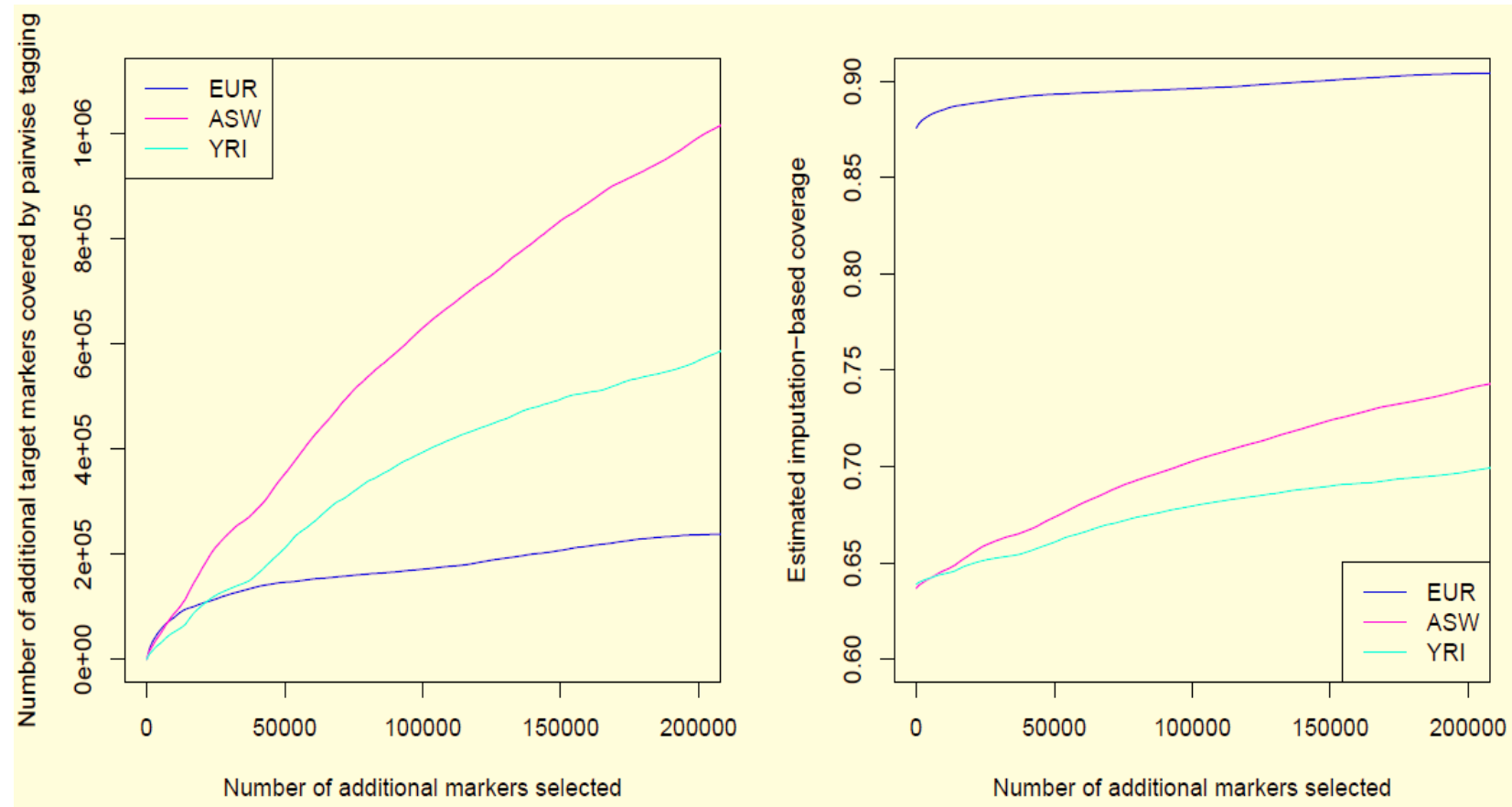

Supplement: Additional file 2: Figure S1. — TxArray transplant-specific modular contents. (PDF 140 kb) [file 13073_2015_211_MOESM2_ESM.pdf]
